# Adherence to growth hormone treatment in the transition age: A prospective observational multicenter study

**DOI:** 10.1210/jendso/bvag022

**Published:** 2026-01-28

**Authors:** Chiara Guzzetti, Paula van Dommelen, Letizia Casula, Marco Cappa, Manuela Caruso, Anna Maria Colao, Mirjana Doknic, Natascia Di Iorgi, Luca Persani, Gabriella Pozzobon, Mariacarolina Salerno, Sandro Loche

**Affiliations:** Pediatric Endocrinology Unit, Pediatric Hospital Microcitemico “A. Cao”, 09121 Cagliari, Italy; Department of Child Health, TNO, 2301 CE Leiden, The Netherlands; Pediatric Endocrinology Unit, Pediatric Hospital Microcitemico “A. Cao”, 09121 Cagliari, Italy; Research Unit for Innovative Therapies in Endocrinology, Bambino Gesù Children's Hospital, IRCCS, 00165 Roma, Italy; Department of Pediatrics, Azienda Policlinico Università di Catania, 95123 Catania, Italy; Endocrinology, Diabetology and Andrology Unit, Department of Clinical Medicine and Surgery, Federico II University of Naples, 80131 Napoli, Italy; Neuroendocrine Department, Clinic for Endocrinology, Diabetes and Metabolic Diseases, University Clinical Center of Serbia, University of Belgrade, 11000 Belgrade, Serbia; Department of Neuroscience, Rehabilitation, Ophthalmology, Genetics, Maternal and Child Health (DINOGMI), Pediatric Clinic, IRCCS Istituto Giannina Gaslini, University of Genoa, 16147 Genova, Italy; Department of Medical Biotechnology and Translational Medicine, University of Milan, Milan, Italy; Department of Endocrine and Metabolic Diseases, IRCCS Istituto Auxologico Italiano, 20149 Milan, Italy; Pediatric Unit, Università Vita-Salute, Milan, San Raffaele Hospital, IRCCS, 20132 Milan, Italy; Department of Translational Medical Sciences, Pediatric Endocrinology Unit, Federico II University of Naples, 80131 Naples, Italy; Pediatric Endocrinology Unit, Pediatric Hospital Microcitemico “A. Cao”, 09121 Cagliari, Italy; Research Unit for Innovative Therapies in Endocrinology, Bambino Gesù Children's Hospital, IRCCS, 00165 Roma, Italy

**Keywords:** growth hormone deficiency, adherence, transition age, IGF-1, Easypod™

## Abstract

**Context:**

Adherence can be an issue in patients taking growth hormone (GH).

**Objective:**

To evaluate adherence to GH treatment during the transition age in patients with permanent childhood-onset GH deficiency (COGHD).

**Methods:**

This is a prospective, multicenter, observational study conducted across 9 European centers. Fifty-one patients aged 15 to 25 years with permanent COGHD, who had reached final height and continued recombinant human GH (0.003-0.02 mg/kg/day), were monitored for 12 months using the Easypod™ device, which provides objective adherence data. Anthropometry, total cholesterol, low- and high-density lipoprotein cholesterol, and insulin-like growth factor 1 (IGF-1) standard deviation score (SDS) were measured at baseline and after 1 year. Patients with ≥9 months of adherence data (*n* = 41) were analyzed.

**Results:**

Twenty-six patients (63%, 16 males) had optimal adherence (≥85%, median, 98%; interquartile range, 91-99%), and 15 (37%, 11 males) had suboptimal adherence (<85%, median, 70%; interquartile range, 47-80%). At baseline, suboptimal adherent patients had greater mean waist circumference (89.2 vs 78.2 cm) and lower mean IGF-1 SDS (−2.2 vs −1.5). After 1 year mean waist circumference (84.8 vs 76.7 cm), mean total cholesterol (185.1 vs 167.0 mg/dL), and mean LDL (111.6 vs 98.5) were higher in the suboptimal adherent group, whereas mean IGF-1 SDS was lower (−1.1 vs −0.2). The mean change in IGF-1 SDS after 1 year was +1.4 vs +0.9 in the 2 groups.

**Conclusion:**

Over 1/3 of patients with permanent COGHD during transition show suboptimal adherence to GH therapy, associated with adverse metabolic markers and persistently lower IGF-1 levels. These findings highlight the importance of adherence monitoring and support targeted interventions to optimize long-term outcomes.

The term transition encompasses a broad spectrum of physical and psychological changes, conventionally defined as beginning in late puberty and concluding with full adult maturation. This period typically extends from mid to late adolescence until ∼6 to 7 years after final height is achieved [1, 2]. Although linear growth ceases during this stage, somatic development continues and individuals reach peak bone mass [1, 2].

Discontinuation of recombinant human growth hormone (rhGH) therapy during the transition phase in patients with childhood-onset GH deficiency (COGHD) has been shown to increase fat mass, decrease muscle mass, reduce bone mineral content, and worsen lipid profiles [3-5]. Conversely, the continuation of rhGH replacement therapy results in increased muscle mass, reduced fat mass, improved bone mineral content and density, and enhanced quality of life [6, 7].

In accordance with current clinical guidelines, patients with COGHD are advised to maintain rhGH therapy throughout the transition years to achieve full skeletal maturation and prevent metabolic abnormalities [8].

The transition from pediatric to adult endocrine care is increasingly recognized as a high-risk stage for patients with chronic conditions, including GHD. The loss of pediatric team support, the necessity to adapt to adult services, and the growing independence of adolescents all contribute to poor treatment adherence. International registry data have indicated that >50% of patients may discontinue GH therapy prematurely, even when persistent GHD is biochemically confirmed [9]. This attrition may compromise attainment of peak bone mass, optimal muscle strength, and long-term cardiovascular protection [1-7]. Psychosocial factors are equally relevant. Adolescents often face competing priorities, academic stress, peer influence, and treatment fatigue. Daily injectable therapy may be perceived as a burden without immediate visible benefits, unlike during the growth years [10].

Previous pediatric studies suggest that 15% to 20% of patients demonstrate poor adherence [11-13], but evidence in transition-age patients remains scarce. Understanding adherence in this age group is critical for designing targeted interventions that safeguard both short- and long-term health outcomes. The advent of electronic injection devices such as Easypod™ provides an opportunity for objective monitoring of adherence, overcoming limitations of self-reported data [14, 15].

This study aims to fill the gap by prospectively evaluating adherence and associated clinical outcomes in young adults with permanent COGHD.

## Materials and methods

This is a prospective, multicenter, observational study in 9 European centers (6 pediatric endocrinology and 3 adult endocrinology, 8 from Italy and 1 from Serbia; from 2019 to 2024). Fifty-one patients aged 15 to 25 years with permanent COGHD were enrolled. Growth hormone deficiency was confirmed with retesting at the end of growth (with insulin tolerance or with arginine plus GH-releasing hormone test) when indicated [8]. Treatment was stopped for 1 to 4 months before retesting and then re-instituted at a dose of 0.01 to 0.02 mg/kg daily. Patients were not retested if they had >3 pituitary hormone deficiencies [1, 2]. When they reached near adult height, the GH dose was lowered to about half the pediatric dose and then titrated according to insulin-like growth factor 1 (IGF-1) concentrations. When they entered the study protocol, they were on treatment with daily rhGH at a dose of 0.003 to 0.02 mg/kg.

Adherence was recorded by the Easypod™ device and calculated as administered/expected doses × 100. Optimal adherence was defined as ≥85% [16]. Anthropometry, total cholesterol (Chol), high-density lipoprotein (HDL) cholesterol, low-density lipoprotein (LDL) cholesterol, and IGF-1 standard deviation score (SDS) were evaluated at baseline and 12 months.

Statistical analysis was performed using analysis of variance, Mann–Whitney, χ^2^/Fisher, and Pearson/Spearman correlation tests. *P* < .05 was considered significant.

The study adhered to the ethical principles outlined in the Declaration of Helsinki and was approved by the Medical Ethics Committee of the Azienda Ospedaliero Universitaria, Cagliari, Italy (Prot. PG/2018/5406). All patients provided written informed consent for participation.

## Results

Forty-one patients (27 males, age 15-25 years) with >9 months adherence data were available for the analysis. Their data are shown in Table 1. Growth hormone deficiency was idiopathic in 21 subjects, congenital in 11, postradiotherapy in 3, and postsurgery in 6. Growth hormone deficiency was isolated in 27 patients. Thirty-five patients underwent retesting, and 6 were not retested. Reasons for withdrawing from the study included side effects [2], withdrawal of consent [2], technical issues with the device [1], lost to follow-up [2], investigator never sent data [1], and unknown reasons [2].

**Table 1:** Clinical characteristics of patients subdivided into optimal (≥85%) and suboptimal (<85%) adherence groups

Characteristic	Optimal adherence (start)	Optimal adherence (1 year)	Suboptimal adherence (start)	Suboptimal adherence (1 year)
*n* (%)	26 (63%)		15 (37%)	
Age (years)	18.5 (2.6)	—	17.3 (2.0)	—
Sex (M/F)	18/8	—	9/6	—
Height SDS	−0.5 (1.1)	−0.5 (1.2)	−0.6 (1.1)	−0.6 (1.1)
Duration of therapy (years)	9.8 (4.0)	—	9.8 (4.1)	—
WC (cm)	78.2 (10.3)	76.7 (8.5)	89.2 (19.2)	84.8 (19.2)
BMI (kg/m^2^)	22.4 (4.2)	22.3 (4.2)	23.5 (8.1)	23.8 (8.0)
Chol (mg/dL)	168.1 (41.6)	167.1 (34.5)	163.5 (40.1)	185.1 (38.5)
LDL (mg/dL)	101.4 (28.7)	98.5 (29.4)	94.9 (32.9)	111.6 (39.2)
HDL (mg/dL)	52.1 (13.0)	52.0 (16.5)	52.6 (15.8)	51.3 (11.6)
IGF-1 SDS	−1.7 (1.7)	−0.2 (1.5)	−2.2 (2.2)	−1.1 (1.5)
Δ IGF-1 SDS		1.4 (1.3)		0.9 (1.8)

Values are mean (SD).

Abbreviations: BMI, body mass index; Chol, total cholesterol; HDL, high-density lipoprotein; IGF-1, insulin-like growth factor 1; LDL, low-density lipoprotein; SDS, standard deviation score; WC, waist circumference.

**Figure 1:**
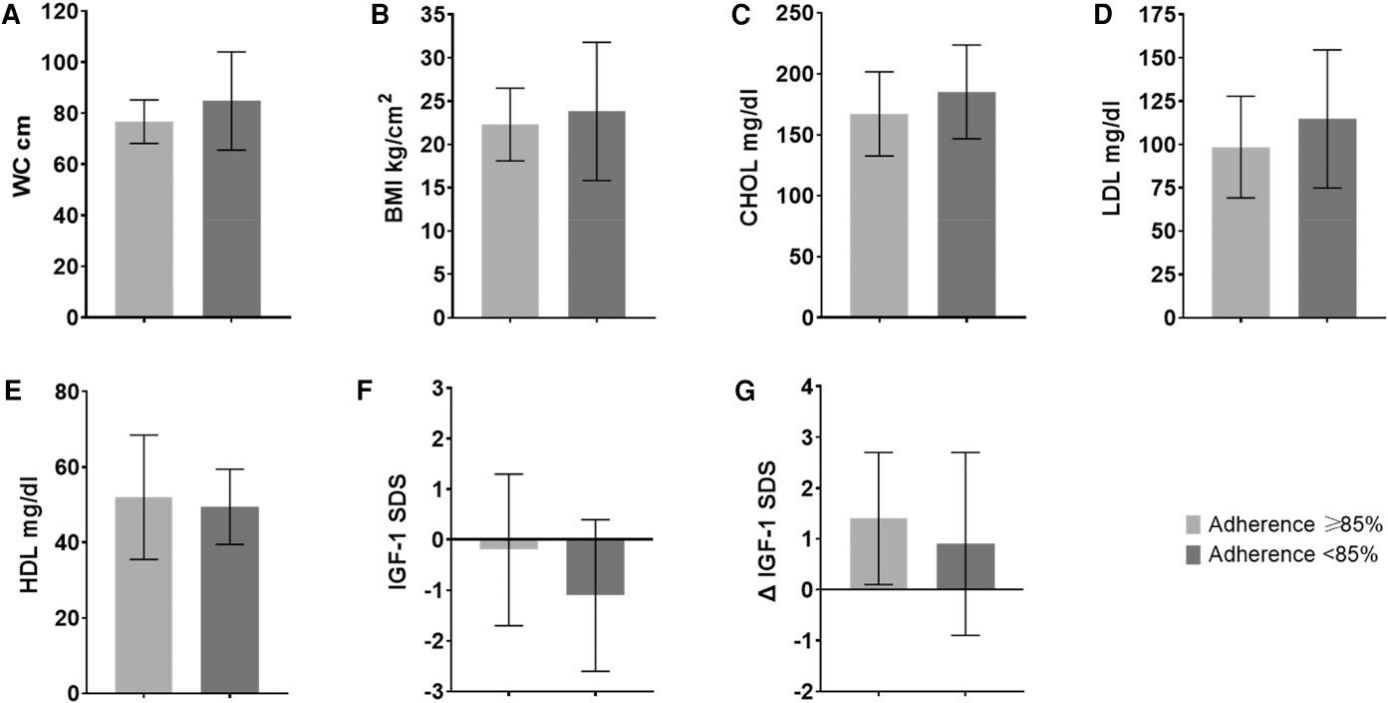
Waist circumference (cm, A), body mass index (kg/m^2^, B), total cholesterol (mg/dL, C), LDL (mg/dL, D), HDL (mg/dL, E), IGF-1 SDS (F), after 1 year observation and change in IGF-1 SDS (Δ IGF-1 SDS, G) between treatment start and 1 year in the 2 adherence groups.

Twenty-six patients (63%, 16 males) had optimal (≥85%) adherence (median, 98%; interquartile range, 91-99%) and 15 (37%, 11 males) had suboptimal (<85%) adherence (median, 70%; interquartile range, 47-80%). The mean (SD) duration of treatment before the study was 9.8 (4.0) years in the optimal adherent group and 9.8 (4.1) years in the suboptimal adherent group. Mean height SDS was similar in the patients with suboptimal adherence (−0.6 ± 1.1 vs 0.5 ± 1.1; Table 1). After 1 year, the mean waist circumference (Fig. 1A) was 76.7 (8.5) and 84.8 (19.2) cm, the mean body mass index (Fig. 1B) was 22.3 (4.2) and 23.8 (8.0) g/m^2^, the mean Chol (Fig. 1C) was 167.0 (34.5) and 185.1 (38.5) mg/dL, the mean LDL (Fig. 1D) was 98.5 (29.4) and 111.6 (39.2) mg/dL, the mean HDL (Fig. 1E) was 52.0 (16.5) and 51.3 (11.6) mg/dL, and the mean IGF-1 SDS (Fig. 1F) was −0.2 (1.5) and −1.1 (1.5) in the optimal and suboptimal adherent groups, respectively. The mean change in IGF-1 SDS (Δ IGF-1 SDS) between treatment start and 1 year later was greater in the optimal (1.4 ± 1.3) than in the suboptimal (0.9 ± 1.8) adherent groups (Fig. 1G). The level of adherence was not correlated with age, sex, duration of treatment, lipids, or the etiology of GHD. There was no difference in the assigned GH dose between the 2 groups.

### Safety

Few AEs were recorded during the study in 4 subjects (2 males and 2 females) and discontinuation of treatment was necessary in 2 patients. No SAE was recorded. The small number of patients and the short-term observation period prevents any statistical evaluation.

## Discussion

This study indicates that adherence must be considered a key therapeutic target during transition. Insulin-like growth factor 1 concentrations increased during GH treatment in both the optimal and the suboptimal adherent patients, although to a lesser extent in the latter. Furthermore, suboptimal adherence was associated with sustained differences in waist circumference and cholesterol. These findings may indicate that IGF-1 alone may not fully capture the metabolic benefits of GH therapy, and that a broader panel of endpoints should be monitored. The American Association of Clinical Endocrinologists recommends fasting lipid profile monitoring at baseline and during follow-up, ideally every 6 to 12 months in GH-deficient adults, including transition-age patients [8]. Furthermore, the results of this study confirm the safety of GH replacement in young adults with GHD as prescribed in routine clinical practice [8].

Beyond its well-established role in promoting growth, GH exerts profound effects on lipid metabolism, glucose homeostasis, body composition, and cardiovascular function [17]. The withdrawal of GH therapy at final height may precipitate metabolic disturbances, including dyslipidemia and increased visceral adiposity, predisposing these young adults to early cardiovascular risk [18]. Previous studies have shown that reinstituting GH replacement in transition patients with confirmed GHD is followed by a reduction in total cholesterol and LDL, a modest or no significant change in HDL, and an improvement in body composition with a reduction in central adiposity [19, 20]. Although differences did not reach statistical significance (probably due to the small number of patients), there was a clear trend for increased cholesterol, LDL, waist circumference, and body mass index values in the patients with suboptimal adherence in our study.

The observed 37% rate of suboptimal adherence is noteworthy, being higher than typical pediatric estimates using the Easypod™ device in the first year of treatment (<15%) [11, 13]. This highlights the vulnerability of the transition period, when patients face new responsibilities and may prioritize education, employment, or social activities over medical routines. Strategies to improve adherence might include digital reminders, educational interventions, psychosocial support, and closer engagement with transition care teams [21]. The fact that the patients know that their adherence is being objectively monitored by an electronic device may serve not only as a monitoring tool but also as a feedback mechanism to empower patients and clinicians to identify adherence barriers [21, 22].

Our findings indicate that adherence remains a critical determinant of treatment outcomes during the transition age. Baseline IGF-1 concentrations were lower in the group with suboptimal adherence. They increased in both optimal and suboptimal adherence groups, but patients with lower adherence maintained lower IGF-1 SDS and higher waist circumference, Chol, and LDL levels, indicating potentially unfavorable metabolic trajectories. The fact that the patients with suboptimal adherence had lower IGF-1 concentrations at baseline may indicate that they were not fully adherent also before entering the study protocol.

Our findings align with previous pediatric adherence studies, where suboptimal adherence was associated with impaired growth outcomes [11, 13, 16, 23]. In young adults, the consequences may manifest primarily as metabolic risks, which could translate into long-term cardiovascular complications if left unaddressed. In this regard, it should be noted that the severity of lipid profile abnormalities in adult patients with GH deficiency is correlated to the severity of GHD [24] and that the patients of our study were all affected by severe permanent GHD.

Despite the small number of subjects, the high rate of suboptimal adherence observed highlights the need for structured transition programs. Educational interventions, digital health tools, and patient-tailored counseling could improve engagement. Interdisciplinary teams that integrate pediatric and adult endocrinologists, psychologists, and nurses are essential to address the multifaceted barriers to adherence. Furthermore, socioeconomic factors, health literacy, and cultural perceptions about chronic therapy should be systematically assessed when planning interventions.

Future research should investigate whether early identification of poor adherence through digital monitoring can enable timely corrective strategies. Large-scale longitudinal studies are needed to evaluate the impact of adherence patterns on cardiovascular, skeletal, and psychosocial outcomes.

Limitations of our study include the relatively small sample size and the observational design, which precludes causal inference. Nonetheless, the multicenter nature and prospective design, coupled with objective adherence measurement, strengthen the reliability of our results. Overall, these findings reinforce the message that adherence monitoring should be integral to endocrine care during transition, with targeted interventions aimed at reducing the proportion of patients at risk.

In conclusion, this prospective multicenter study demonstrates that over 1/3 of young adults with permanent COGHD exhibit suboptimal adherence to rhGH therapy during the vulnerable transition period. Suboptimal adherence is associated with unfavorable metabolic profiles, including increased waist circumference, Chol, and LDL levels, despite similar IGF-1 improvements compared with adherent patients. These findings emphasize that IGF-1 alone may inadequately reflect the metabolic benefits of GH therapy. Given the known long-term risks of untreated GHD, particularly cardiovascular complications, systematic objective adherence monitoring should be integrated into transitional endocrine care. Multidisciplinary approaches, including digital health interventions and psychosocial support, are essential to improve adherence and optimize health outcomes. Ultimately, improving adherence during transition may reduce lifelong morbidity and healthcare costs for patients with COGHD.

## Data Availability

Some or all datasets generated during and/or analyzed during the current study are not publicly available but are available from the corresponding author on reasonable request.

## Abbreviations

COGHD: childhood-onset GH deficiency
GHD: growth hormone deficiency
HDL: high-density lipoprotein
IGF-1: insulin-like growth factor 1
LDL: low-density lipoprotein
r-hGH: recombinant human GH
SDS: standard deviation score
